# N-Glycoproteome of E14.Tg2a Mouse Embryonic Stem Cells

**DOI:** 10.1371/journal.pone.0055722

**Published:** 2013-02-06

**Authors:** Bingyun Sun, Li Ma, Xiaowei Yan, Denis Lee, Vinita Alexander, Laura J. Hohmann, Cynthia Lorang, Lalangi Chandrasena, Qiang Tian, Leroy Hood

**Affiliations:** 1 Institute for Systems Biology, Seattle, Washington, United States of America; 2 Department of Chemistry, Simon Fraser University, Burnaby, British Columbia, Canada; Institute of Molecular and Cell Biology, Singapore

## Abstract

E14.Tg2a mouse embryonic stem (mES) cells are a widely used host in gene trap and gene targeting techniques. Molecular characterization of host cells will provide background information for a better understanding of functions of the knockout genes. Using a highly selective glycopeptide-capture approach but ordinary liquid chromatography coupled mass spectrometry (LC-MS), we characterized the N-glycoproteins of E14.Tg2a cells and analyzed the close relationship between the obtained N-glycoproteome and cell-surface proteomes. Our results provide a global view of cell surface protein molecular properties, in which receptors seem to be much more diverse but lower in abundance than transporters on average. In addition, our results provide a systematic view of the E14.Tg2a N-glycosylation, from which we discovered some striking patterns, including an evolutionarily preserved and maybe functionally selected complementarity between N-glycosylation and the transmembrane structure in protein sequences. We also observed an environmentally influenced N-glycosylation pattern among glycoenzymes and extracellular matrix proteins. We hope that the acquired information enhances our molecular understanding of mES E14.Tg2a as well as the biological roles played by N-glycosylation in cell biology in general.

## Introduction

Embryonic stem (ES) cells exhibit unique properties of self-renewal and pluripotency, possessing broad applications in developmental biology and regenerative medicine [1], [2]. Mouse ES (mES) cells are valuable tools to produce genetically modified mouse strains through gene targeting and gene trapping techniques for studies in functional genomics and biomedical research. Molecular characterization of host mES cells provides background information for a better functional understanding of the knockout gene(s). Towards this end, we here focus on deciphering the N-glycoproteome of E14.Tg2a, one of the most popular host cell lines used for gene targeting and gene trapping [3].

The mES cell line, E14.Tg2a, derived by Hooper *et al.* from 129/OLA in 1987 [4] was originally developed as a mouse model of Lesch-Nyhan disorder with a deficiency of hypoxanthine phosphoribosyltransferase (HPRT). E14.Tg2a cells grow fast and steadily in both feeder and feeder-free systems, and produce higher success of germ-line transmitting chimera than mES cells derived from BL6 strains [5]. Therefore, E14.Tg2a is an ideal system for genetic engineering. To date, at least 29,000 transgenic mice and mES cell lines have been derived from E14.Tg2a (Dr. Richard Baldarelli, Mouse Genome Informatics, The Jackson Laboratory, personal communication).

Lesch-Nyhan syndrome is a metabolic disorder hallmarked by hyperuricemia, mental retardation and self-mutilation [6]. The pathology of the E14.Tg2a host potentially complicates the use of this system in deciphering target gene functions. Therefore, there is a need to elucidate molecular details of this cell line itself in building a well-understood genomic background. Even though several high-throughput molecular characterizations have been carried out to E14 mES cells, little attention has been directed towards the E14.Tg2a subclone (except for the proteomic characterization of the chromatin remodeling complex conducted by Ho L. *et al.* in 2009) [7]. Seegmiller *et al.* stated in 1967 [6] that Lesch-Nyhan syndrome is the first example of an abnormal compulsive behavior raised by a specific enzymatic defect; and it is also the first demonstrated enzymatic defect in purine metabolism in a neurological disease. Thus, it is also interesting to elucidate the protein makeup of E14.Tg2a mES cells from pathologic and metabolic viewpoints.

In stem cells, the choice of proliferation and differentiation is largely regulated by the interaction between cell surface proteins and cells’ microenvironment, i.e. the stem-cell niche. Both the cell surface and the niche are rich in glycoproteins, especially N-linked glycoproteins. N-glycosylation is a co-translational modification that takes place at the ER, and functions importantly in protein folding, stabilization, membrane trafficking, and interaction with other molecules. The complete removal of N-glycosylation from all cellular proteins is embryonically lethal [8], [9], and the aberrant N-glycosylation on individual proteins can cause severe birth defects, including but not limited to the congenital disorder of glycosylation (CDG) as well as lysosomal storage diseases [10]. N-linked glycoproteins reside specifically at the outer plasma membrane, in the extracellular milieu, secretory channel (i.e. ER and Golgi apparatus) and endocytic pathway (lysosomes and endosomes) [11]. The external facing of N-linked polypeptides at the cell-outer membrane makes these proteins ideal candidates as markers of stem cells, and most known ES-cell surface markers are indeed N-glycoproteins including Thy1 (CD90), c-kit (CD117), Lrp2 (endoglin), Prom 1 (CD133) and neural cell adhesion molecule (NCAM) [12], [13]. In Lesch-Nyhan syndrome, the impaired purinergic metabolism is initiated from cell-surface purinergic receptors which are also modified by N-glycans [14]. Hence, decoding the N-glycoproteome of E14.Tg2a will benefit the research and application of mES cells, as well as the studies of the pathophysiology of Lesch-Nyhan syndrome.

A comprehensive characterization of the N-glycoproteome is, however, technically challenging. N-glycans comprise the most complex and diversified structures among all known protein post-translational modifications; and membrane-bound N-glycoproteins inherit the challenges of membrane-protein studies: the low solubility in aqueous solution and the low abundance [15], [16]. To conquer these challenges, researchers have separated glycan-centric glycomics from protein-centric glycoproteomics in high-throughput analyses [17]–[20]. For protein-centric N-glycoproteomics, the enrichment of N-glycoproteins is often necessary for sensitive analyses, and several techniques have been developed for this purpose, including the lectin affinity enrichment, boronic acid and hydrazide based chemical enrichments among other chemical or physical methods [17], [21].

To date, N-glycoproteomics has been carried out to mES cells [22], yet no effort has been focused on the E14.Tg2a subclone. To characterize the N-glycoproteome of E14.Tg2a, we used a previously developed N-glycopeptide capture strategy, a technique optimized for membrane N-glycoproteins [23]. Using conventional liquid chromatography (LC) and low-end mass spectrometer (MS), we cataloged the low-abundance N-glycoproteins and their glycosylation patterns in E14.Tg2a cells. The subsequent data analyses allowed us to discover some novel structural and functional relations among membrane proteins. All proteomic data presented here has been deposited in the publically available Peptide Atlas database (http://www.peptideatlas.org/) [24].

## Materials and Methods

All the chemicals were purchased from Thermo Fisher if not specified. The Bradford kit, sodium periodate (Affi-Gel oxidizer, cat. 153-6055), and hydrazide resin (Affi-Gel, cat. 153-6047) were obtained from Bio-Rad. PNGase F was from New England Biolabs, and sequencing grade trypsin (cat. V5111) was from Promega. RapiGest and C18 columns were from Waters, and ZipTip C18 (cat. ZTC18) was from Millipore. Cell culture medium (GMEM), ß-mercaptoethanol, and protease inhibitors were from Sigma, and leukemia inhibitory factor (Lif) was from Chemicon. All other cell culture reagents were from Invitrogen.

### Cell Culture and the Preparation of Crude Membrane Fraction

Both E14.Tg2a derived from *Mus Musculus* strain 129/Ola^3^ (obtained from The Wellcome Trust Sanger Institute) and D3 (obtained from American Type Culture Collection, cat. CRL-11632) mES cell lines were maintained on 0.1% gelatin-coated tissue culture dishes in GMEM medium supplemented with 2-mM glutamine, 1-mM sodium pyruvate, nonessential amino acids, 10% fetal bovine serum, 50-µM ß-mercaptoethanol, and 1000 U/ml leukocyte inhibitory factor.

The crude membrane fraction of ES cells was prepared according to previous procedures [25]. In brief, cultured cells were washed with phosphate buffered saline (PBS) and removed from culture dishes by scraping. After centrifugation at 1,500 rpm for 5 min at 4°C, cells were re-suspended in 10-ml hypotonic buffer (20-mM Tris-HCl pH 7.4, 10-mM MgCl_2_, 10-mM CaCl_2_) containing protease inhibitors, and incubated on ice for 15 min prior to homogenization. Cells were lysed by Dounce homogenization (∼50 strokes), and the lysis was confirmed by trypan-blue staining. The microsomal fraction was obtained by differential centrifugation first at 4°C and 3,000 *g* for 15 min, and then at 4°C and 100,000 *g* for 2 h (Beckman Coulter L8-70M Ultracentrifuge). The final pellet containing the plasma membrane and endomembrane systems was stored at −80°C.

### Glycopeptide Capture

The microsomal fraction was dissolved in a denaturing buffer containing 0.5% RapiGest® and 8-M urea, and digested into peptides prior to glycopeptide capture using a previously described protocol [23] with minor modifications. In a typical preparation, about 0.5–0.8 mg microsomal protein was obtained from ∼3×10^8^ cells. Proteins were denatured, alkylated, and the sample solution was diluted at least ten times before the trypsin digestion step. The detergent, RapiGest, was removed by degrading the digest at pH ∼1 for 1 h at 37°C, and the developed precipitation was removed by centrifugation at 120,000 *g* for 25 min. The pH of the supernatant was brought to ∼3 prior to desalting through a 1-ml Sep-Pak C18 cartridge. The digestion efficiency was verified by SDS-PAGE (i.e. sodium dodecyl sulfate polyacrylamide gel electrophoresis).

The capture and recovery of N-glycopeptides have been described previously in detail [23]. Briefly, the digested peptides were oxidized by incubating with 10 mM (final concentration) freshly prepared sodium periodate in 200 µl coupling buffer at pH 5.0 in the dark for 1 h, and then quenched with 20-mM (final concentration) freshly prepared sodium sulfite (pH 4–5) for 10 min. The glycosylated peptides were coupled to ∼100-µl hydrazide resin equilibrated in 500-µl coupling buffer, and incubated overnight at 37°C with end-over-end rotation. A series of post-coupling washes were applied sequentially with deionized water, 100% methanol and 80% acetonitrile to remove un-bound peptides. The captured N-linked glycopeptides were selectively released from the resin by incubation with 2-µl PNGase F at 37°C overnight with end-to-end rotation.

### Chromatographic Separation and MS Identification of Glycopeptides

Aliquots of released peptides were used for both direct reverse-phase split nanoflow liquid chromatography (rp-nano-LC) and tandem MS analysis (MS/MS), or further fractionated by strong-cation exchange chromatography (SCX) prior to rp-nano-LC-MS/MS. For SCX, peptides were separated on a cartridge (Applied Biosystems) at a flow rate of 50 µl/min with step gradients of 25, 75, 125, 175, and 1000-mM KCl in a 10-mM potassium phosphate and 25% acetonitrile buffer at pH 3.0. Both the flow through and eluents were collected and desalted on C18 columns prior to rp-nano-LC-MS/MS analysis.

For MS/MS analysis, a linear-quadrupole ion-trap LTQ mass spectrometer (Thermo Scientific, Waltham, MA) was used, as detailed in File S1 [23]. The obtained mass spectra were converted to mzXML format using software developed in-house; spectra with fewer than six ions and intensities less than 100 were discarded [26], [27]. The converted mzXML files were searched against the ipi.MOUSE.v3.82.fasta database supplemented with common contaminating sequences (trypsin and keratin) by SEQUEST (v.27, (c) 1993, Thermo Finnigan). We took 218 entries of human keratins from the Human IPI database [28]. The mass tolerance for precursor mass was ±3.0, and the mass tolerance for MS/MS was ±0.5 [29]. For search parameters, we used carbamidomethylated cysteine (+57) as the fixed modification, and oxidized methionines (+16) and asparagine-to-aspartic acid (+1) conversion during PNGase F enzymatic cleavage as variable modifications. The PeptideProphet™ and Protein Prophet™ algorithms with constraints of at least one tryptic end, two possible miss cleavages, and N-glycosylation were used to evaluate the quality of peptide and protein identification [28], [30].

Protein IPIs identified by the same set of peptides were listed as degenerative entries. The first IPI was used in translation to gene symbols and Entrez gene IDs for comparison with other datasets. We also estimated the relative protein quantity using spectra counting [31]. In detail for each protein, we computed the sum of peptide spectra counts from all MS results, and normalized it with the number of detected N-glycans (the N-glycan stoichiometry) for protein quantity. The orthologous proteins among different species including *Homo Sapiens*, *Caenorhabditis Elegans*, *Drosophila Melanogaster*, and *Danio Rerio* were obtained through HomoloGene from NCBI (http://www.ncbi.nlm.nih.gov/sites/entrez?db=homologene). Transmembrane domain prediction was obtained by TMHMM Server v. 2.0 (http://www.cbs.dtu.dk/services/TMHMM/). GoMiner (http://discover.nci.nih.gov/gominer/index.jsp) was used for the enrichment analysis of Gene Ontology.

### Immunoassay Validation of Selected CD Proteins

For Western blot, each sample was loaded at equal protein quantity to a SDS-PAGE gel, and immunoblotted using commercial primary antibodies against Cd9, Cd36, and Cd55 (Santa Cruz Biotechnology, Santa Cruz, CA) and horseradish peroxidase-coupled secondary antibodies. GAPDH was used as a loading control. Immunoreactive proteins were visualized by enhanced chemiluminescence. For flow cytometry, both D3 and E14.Tg2a cells were incubated with FITC-conjugated antibodies or isotype controls in a staining buffer (PBS/0.5% BSA/2 mM EDTA) for 30 min at 4°C. Cells were then washed with the staining buffer and resuspended in PBS. Flow cytometry was performed using FACScalibur (Becton Dickinson) and the data were analyzed by the Summit software (Beckman Coulter). The anti-CD230 antibody was from Abcam, and antibodies against Cd133 and Cd90 were from BD Biosciences.

## Results

### Low Cost Shotgun Glycopeptide-capture Strategy

We used a rugged N-glycoproteomic strategy and analyzed E14.Tg2a mES cells, in which no high-end LC and MS were employed. To eliminate the interference of abundant cytosolic proteins, and to minimize the use of the expensive enzyme and acid-cleavable detergent for proteolysis, we enriched the cell microsomal fraction prior to tryptic digestion [25]. The end product of our process was deglycosylated N-linked peptides with glycans removed by an endoglycosidase, PNGase F. The N-glycosylation site was determined by a consensus sequence, NXS/T (sequon), with X being any amino acid except proline [32], [33]. The enzyme PNGase F converts the asparagine residue in the sequon to an aspartic acid [34] and creates a 0.98-Da shift to the peptide mass. Due to the limited resolution of our LTQ configured for high throughput analyses, this mass shift was not distinguishable. However, the high selectivity of our enrichment method ensured a high yield of sequon-containing peptides (more than 90% with confident probability p>0.9), which is significantly higher than those from samples processed without the enrichment step (less than 1%).

One drawback of using less accurate MS detection is when two or more sequons coexist in a single peptide, the exact site of glycosylation is difficult to determine. Fortunately, our data showed that these cases were rare (i.e., ∼ 5.6% of all the identified peptides). Therefore depending on the application, ordinary MS such as the LTQ as we demonstrated here is capable to catalog N-glycoproteomes when higher-end MS instruments are not available.

### The N-glycoproteome

In total, we carried out three biological replicates (separated cultures), and all the samples including the prefractionated samples were analyzed three times (technical replicates) by LC-MS. The detailed evaluation of sample processing and LC-MS analyses is included in the Supplementary Material (Figure S1 in File S1). Table S1 summarizes both the original proteome, and the N-glycoproteome after a removal of both non-N-glycoproteins (no sequons) and single hits, in which potential false positives are prone. In total, we identified 468 IPI glycoproteins with a probability greater than 0.9 and an error rate less than 1.0% [30]. Table S2 lists 405 unique glycoproteins that were successfully translated to gene IDs and gene symbols by PIPE (Protein Information and Property Explore) [35], a web-based program for the management and functional analysis of proteomic data.

Using protein spectra counts, we quantified the relative abundance of identified Entrez gene in Table S2, and empirically defined three quantity levels, i.e. “low”, “medium” and “high”, based on the criteria of “<5, 5 ∼ 12, and >12 spectra” respectively. To put these relative quantities into prospective, we used leukemia inhibitory factor receptor (Lifr) – a mES cell surface marker with a known expression of ∼ 100 copies/cell [36] – as a reference to link the relative quantity with the absolute value. Because Lifr was identified as 4.4 protein spectra and the minimum protein spectra in our results is 1, our detection sensitivity can be roughly estimated as below 100 protein copies. Based on our definition, about 40% of all identified N-glycoproteins are of low abundance, which agrees with the low abundance knowledge of membrane proteins.

### Characterization of the Obtained N-glycoproteome

**Figure 1:**
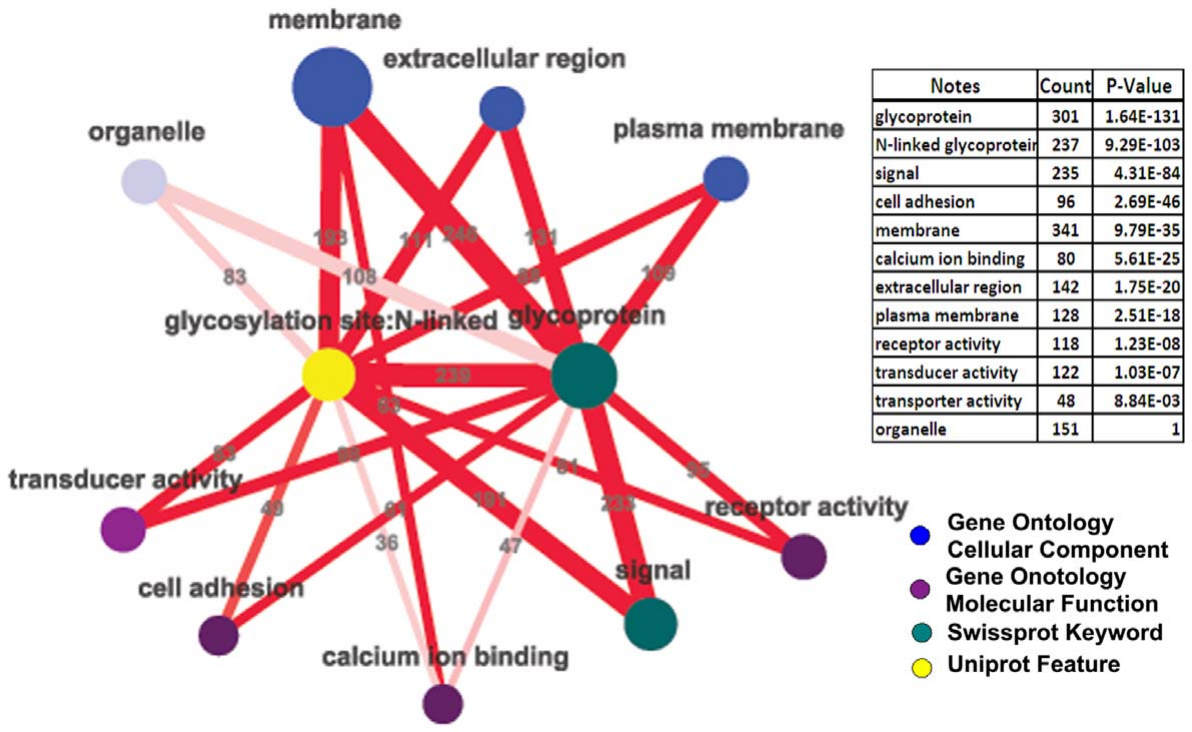
Summary of highly represented protein classes in the N-glycoproteome derived from multiple annotation sources. The color of the nodes indicates different data sources. The size of the nodes and the width of the edges represent the number of proteins that are listed in the table or labeled in the figure, and the color depth of both the nodes and the edges represents the correspondent enrichment p values.

We annotated and cataloged the obtained N-glycoproteins using several well-known protein databases including Gene Ontology (GO), SwissProt, and UniProt. Similar to DAVID (Database for Annotation, Visualization, and Integrated Discovery) [37], [38], we grouped the enriched annotations. Instead of displaying the results as a list, we visualize them using Cytoscape [39], a graphical network analysis platform, as shown in Figure 1. Our network representation not only displays the catalogs (notes) and the corresponding enrichment factors (p value represented by the tinting color), but also highlights the overlapping relations among notes (edges) and their enrichment factors (color). The outstanding protein catalogs are those closely related to cell membranes, such as the “membrane”, “plasma membrane”, “extracellular region”, “receptor activity”, “signal”, “cell adhesion” and “transducer activity”. All these notes are highly overlapping to each other represented by the thick edges connecting them as well as the dark edge color. Notably, a large number of proteins in Table S2 are not included in the databases we used, indicating the incompleteness of these databases.

**Figure 2:**
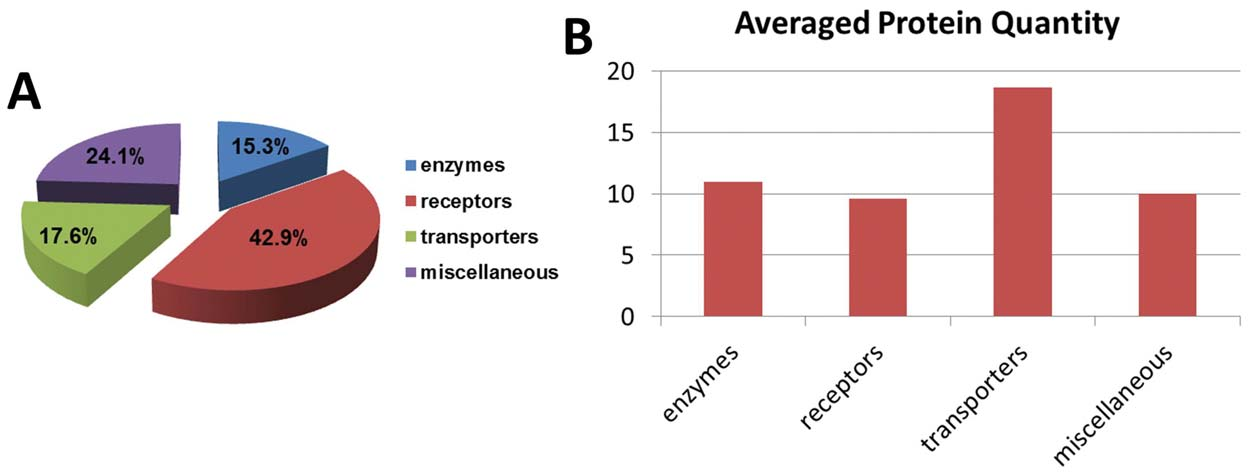
Functional classification of N-glycoproteome based on the definition of Almen *et al*. [**40**] **.** Protein classes are mutually exclusive. A. Percentage distribution of each protein class. B. Average protein spectra counts of each protein class.

To avoid the annotation redundancy, we also classified our results using a mutually exclusive cataloging scheme provided by Almen *et al.* [40] on predicted membrane proteins from the sequence of genome, in which four major functional groups are defined: receptors, transporters, enzymes and miscellaneous (Figure 2 and Table S3). Because Almen *et al.*’s results were obtained from the human genome, we first convert their results into mouse orthologs. To ensure the transmembrane (TM) domain has been preserved from human to mouse, we performed a TMHMM prediction on mouse orthologs and the results agreed well with those of Almen’s (Table S3). Proteins resolved from such cataloging are presented in a pie chart (Figure 2A) and a histogram (Figure 2B) respectively. Qualitatively (Figure 2A), receptors are the most diversified protein class occupying 42% of all annotated protein species; this percentage is twice as high as that of other protein classes such as transporters (17%) and enzymes (15%). Our experimental findings are consistent with the computed results by Almen *et al.* from genomic sequences [40]. Quantitatively in Figure 2B, transporters constitute the most abundant membrane-protein class with an average quantity of 19 spectra (high), almost doubling the quantities of the others, such as receptors (10 spectra) and enzymes (11 spectra).

Besides transmembrane proteins, i.e. 60% (based on the overlap with Almen’s results in Table S3), many other interesting membrane and membrane associated proteins are also present in our dataset. Among proteins that are lacking the predicted TM domain, many are glycophosphatidylinositol (GPI) anchored membrane proteins (e.g., Car4, Thy1, Msln, Gpc1, Vnn1, Raet1b, Raet1a, Rab23, Bst1, Tex101), extracellular matrix components such as protocadherins, cadherins, collagens, laminins, fibronectins, extracellular enzymes (e.g., Sulf1, Ggh, Smpdl3b), secreted molecules (e.g., Scube3, Pxdn, Sema3e, Sema7a, Ltbp1, Loxl2, Fstl1, Tect1, Pltp), growth factors and cytokines (e.g., Bmp1, Grn, Mfge8). We also identified many functionally important proteins at cell surface with membrane association yet to be determined experimentally. Some of these proteins, such as Nomo1 [41], form complexes with membrane proteins and participate in nodal signaling during vertebrate development; others like Tulp1 [42] plays potentially critical roles in photosensing. Taken together, our N-glycoproteome provides experimental supports to many membrane proteins as well as membrane associated proteins that cannot be predicted by TM analyses.

### Comparison of Our N-glycoproteome to Other Publicly Available Cell-surface Proteomes

**Figure 3:**
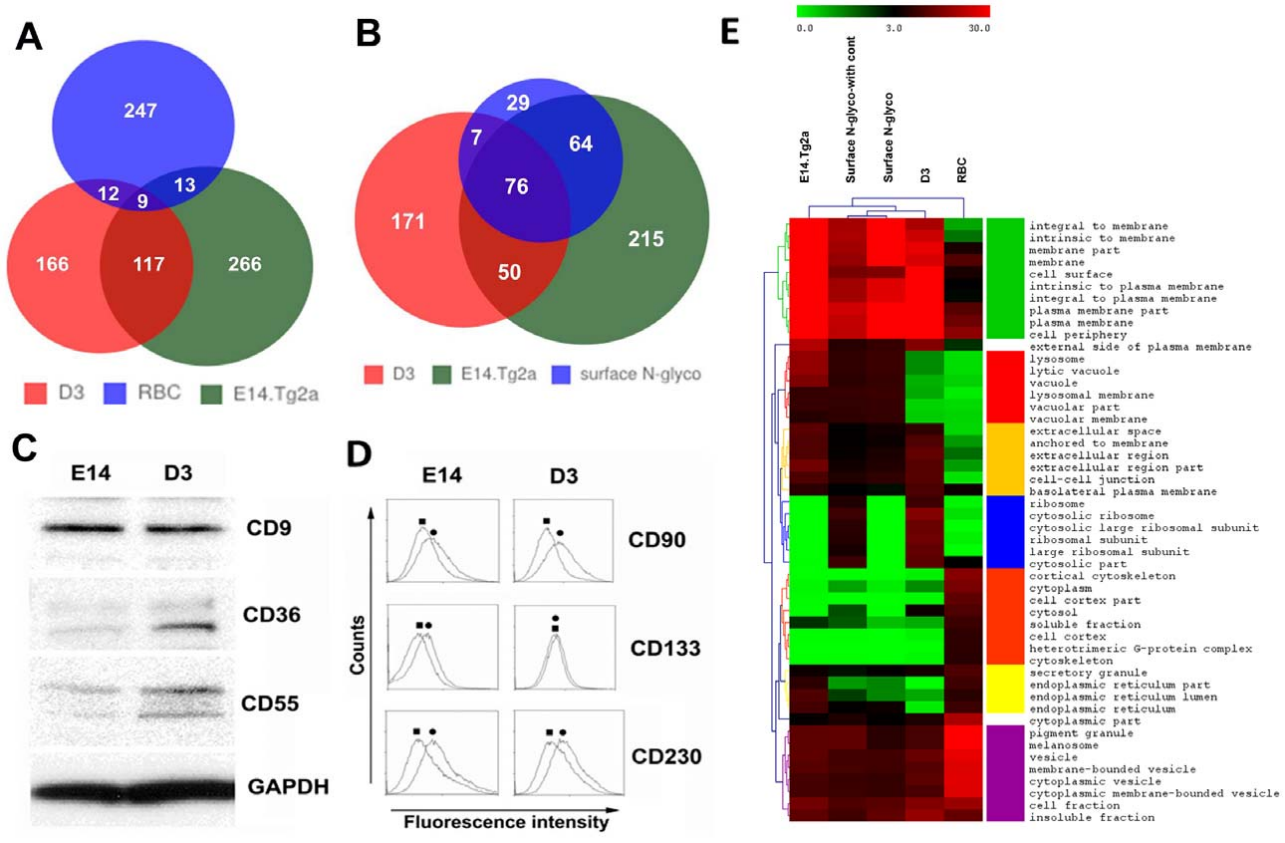
Comparison between our N-glycoproteome (E14.Tg2a), and the other membrane proteomes. The other membrane proteomes include the surface N-glycoproteome without contaminating proteins (surface N-glyco) and the biotinylated cell-surface proteome (D3) [43] of mES cells as well as the red blood cell-surface proteome (RBC) [44]. A) The Venn diagram of D3, E14.Tg2a, and RBC proteomes. B) The Venn diagram of D3, E14.Tg2a, and surface N-glyco proteomes. C) Western blotting validation of CD proteins identified in D3 but not E14.Tg2a proteome, GAPDH is the loading control. D) Flow-cytometry analysis CD proteins identified in E14.Tg2a but not D3 proteome. E) Heatmap of cellular localization Gene Ontology enrichment results among all the compared results, and the color denotes the –log_10_(Pvalue). We also include the surface N-glycoproteome with contamination (surface N-glyco-with cont.) in this comparison.

Because of the close relationship between N-glycoproteins and surface proteins, we analyzed the difference and similarity of our results with three publically available cell-surface proteomes: the mES D3 cell-surface proteome [43] obtained by using an avidin enrichment of biotinylated outer-surface proteins, the mES cell-surface N-glycoproteome [22] obtained by using a selective capture of plasma membrane glycoproteins, and the red-blood-cell (RBC) membrane proteome [44] obtained by using differential centrifugation. Both the heatmap of GO enrichment and the Venn diagram were used to evaluate results as shown in Figure 3.

In Figure 3A, the Venn diagram displays a higher overlap among mES cells than those between ES cells and RBCs. For example, ∼ 30% of the proteins of E14.Tg2a N-glycoproteome are also found in D3 surface proteome, but only 5% in RBC membrane proteome. The overlapping proteins between our results and the surface N-glycoproteome (without contamination) is as high as 79% of the surface N-glycoproteome (Figure 3B); among them, the sites of N-glycosylation overlap 78% of the surface N-glycoproteome and the quantity of these commonly identified proteins are also comparable as shown in Figure S3 in File S1. We have also used these surface proteome datasets to validate our findings on the qualitative and quantitative comparison of protein classes based on the definition of Almen *et al.* and details are summarized in the File S1.

As the mES cell types used for these comparisons are different, we wonder whether the molecular discrepancy in the results can be used as biomarkers to distinguish the two mES cell subtypes. Using Western blot and fluorescence aided flow cytometry, we selectively tested CD (cluster of differentiation) proteins (Table 1) that are uniquely identified by the avidin-biotin membrane enrichment and our glycopeptide capture in D3 and E14.Tg2a cells respectively (Figure 3 C & D). First of all, our results validated the identification of all the proteins in both studies. Secondly using flow cytometry, we also confirmed the surface localization of the selected proteins in our results. However, all the tested proteins, except Cd133, showed expression in both cell lines. Therefore, the observed discrepancy between two proteomes is likely not contributed by the cell subtype difference but by the techniques used. In fact, both cell lines were derived from the same *Mus Musculus* strain 129 [45], thus, it is reasonable to observe similar protein profiles from these cells. The inability for both methods to identify commonly expressed proteins denotes the incomprehensiveness of resolved proteomes – a challenge faced by the entire proteomics field. For proteomics research, we are truly at “the end of the beginning” instead of “the beginning of the end” [46].

**Table 1:** Differentially identified mESCs CD proteins in our mES E14.Tg2a N-glycoproteome and mES D3 surface proteome [43].

CD name	Gene symbol	TMs	Glycoprotein spectra counts	Detected N-glycans	Sequons
**N-Glycoproteome**					
**CD107b**	**Lamp2**	1	1.7E+01	9	17
**CD108**	**Sema7a**	1	3.5E+00	2	5
**CD116**	**Csf2ra**	1	3.5E+00	2	8
**CD133**	**Prom1**	5	8.0E+00	5	8
**CD147**	**Bsg**	1	1.7E+01	3	4
**CD155**	**Pvr**	1	1.2E+01	2	11
**CD157**	**Bst1**	1	3.0E+00	1	4
**CD166**	**Alcam**	1	9.0E+00	2	8
**CD201**	**Procr**	1	1.0E+01	1	5
**CD202b**	**Tek**	2	4.0E+00	2	12
**CD230**	**Prnp**	2	1.2E+01	1	2
**CD276**	**Cd276**	1	5.0E+01	4	4
**CD280**	**Mrc2**	1	5.0E+00	4	14
**CD315**	**Ptgfrn**	1	1.1E+01	4	4
**CD316**	**Igsf8**	1	2.5E+01	3	4
**CD317**	**Bst2**	1	6.5E+01	1	2
**CD318**	**Cdcp1**	1	1.6E+00	5	14
**CD322**	**Jam2**	1	1.6E+01	2	5
**CD326**	**Tacstd1**	1	6.1E+01	2	2
**CD331**	**Fgfr1**	1	1.2E+01	2	9
**CD339**	**Jag1**	1	3.0E+00	2	12
**CD49a**	**Itga1**	2	5.1E+00	7	23
**CD66a**	**Ceacam1**	1	1.9E+01	8	16
**CD90**	**Thy1**	0	1.9E+01	1	4
**Surface Proteome**					
**CD140a**	**Pdgfra**	2	–	–	10
**CD156b**	**Adam17**	1	–	–	8
**CD249**	**Enpep**	1	–	–	9
**CD292**	**Bmpr1a**	1	–	–	3
**CD332**	**Fgfr2**	2	–	–	10
**CD36**	**Cd36**	2	–	–	9
**CD55**	**Cd55**	1	–	–	2
**CD61**	**Itgb3**	1	–	–	6
**CD81**	**Cd81**	4	–	–	1
**CD9**	**Cd9**	4	–	–	1

Regardless of the small overlap in the Venn diagram, the enrichment analyses of Gene Ontology (GO) localization and function of the datasets yield a high degree of similarity. We used a non-supervised hierarchical classification and a heatmap offered by MEV (MultiExperiment Viewer) [47] to analyze and display the enrichment results obtained from GeneGO (GeneGo Int.), a bioinformatic software solution to system biology. Figure 3E interprets the cellular location of the differentially identified proteins indicated in Figure 3A, and the results disclose not only the technical differences and similarities but also the underlying biological difference.

As anticipated, all the compared surface proteomes comprised mainly plasma-membrane proteins. Yet, membrane-related GO terms – “membrane”, “membrane part”, “intrinsic to membrane” and “integral to membrane” *etc.* – are more pronounced in the N-glycoproteome than other comparing proteomes. Besides plasma-membrane proteins, our N-glycoproteome is also rich of membrane-bound organelles including “lysosomes”, “vacuoles”, and “endoplasmic reticulum” (ER). These results reflect the difference in the experimental design, because our method does not exclude glycoproteins from other membrane-bound organelles. The high enrichment of ribosomal proteins in both the D3 surface proteome [43] and the surface N-glycoproteome with contamination [22] also agrees with findings in the respective publications. For the surface N-glycoproteome, [22] Wollscheid *et al.* have specifically listed these cytosolic proteins as contaminants, and the removal of these contaminants gives similar enrichment results as those of our dataset (Figure 3E). Certain differences are also reflecting the diversified cell biology. For example, in RBC dataset, vesicle-related GO terms were more enriched than those of mES-cell datasets, whereas extracellular components were the opposite. Such findings are supported by the enhanced vesicular structures and the circulating nature that are unique to mature red blood cells [48], [49] but not to stem cells. Thus, the enrichment analysis applied here appears to be reliable and informative.

### Comparison of Membrane Proteins to Genomic and Whole-cell Proteomic Analyses

**Figure 4:**
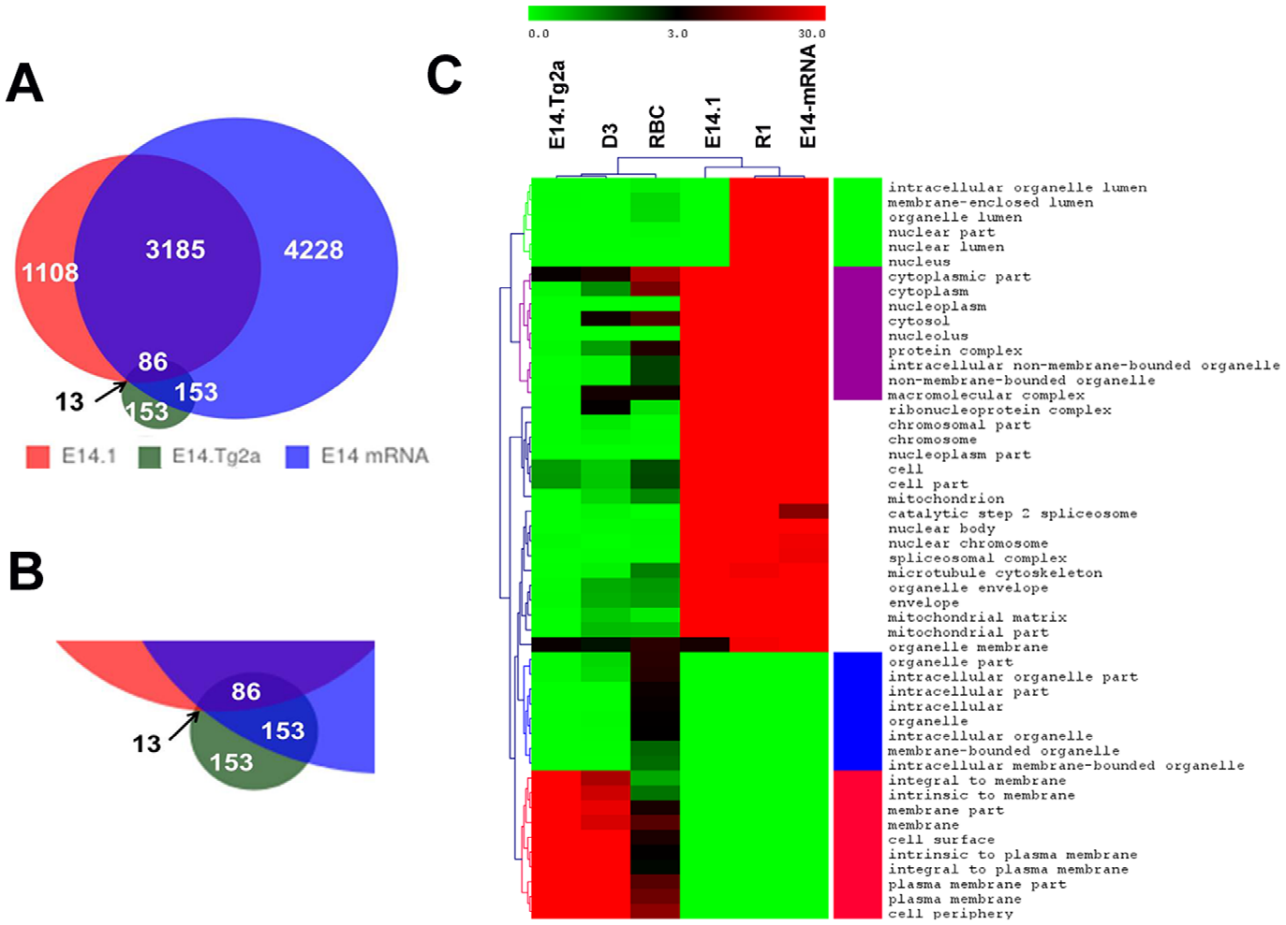
Comparisons among our N-glycoproteome, the overall proteome and transcriptome of mES cells. A) A Venn diagram of identified proteins from our N-glycoproteome (E14.Tg2a), the total proteome of E14.1 mES (E14.1) [50] and the transcriptome (E14 mRNA) [51]. B) The enlarged image of the overlapping region of the three datasets. C) The heatmap of Gene Ontology enrichment results of cellular localization among all the compared datasets, and the color of cells denotes the –log_10_(Pvalue).

To disclose the advantages of using N-glycoproteomics to identify membrane-associated gene products, we further compared three membrane proteomes of E14.Tg2a, D3 and RBC with genomic and whole-cell proteomic results of E14 mES cells, including a whole-cell proteome (Ji Q. R. 2011 [50], 4581 total proteins) and a transcriptome (7,652 genes) obtained by high-coverage gene-expression profiling (HiCEP) [51]. Figure 4 **(**similar as Figure 3) summarizes the comparison results among all the datasets. The Venn diagram (Figure 4A) shows that proteins identified uniquely in our dataset are less abundant (on average) than those identified commonly. The information discovered from the heatmap of GO enrichment results (Figure 4B) matches again the existing knowledge; for example cytosolic proteins are enriched in global analysis but not in membrane targeted studies. We are surprised that not only the overall proteome [52] but also the transcriptome has under-represented membrane proteins [12].

A further functional analysis of uniquely identified proteins in our glycoproteome by GoMiner® reviewed important activities of transmembrane receptor protein kinases and tyrosine kinases, activities of peptidases, and bindings of growth-factors, cell adhesion-molecules, and carbohydrates to name a few (Table S4). Many of these uniquely identified proteins such as receptor tyrosine-kinase like orphan receptor (Ror2), [53]–[55] purinergic receptors (P2rx7) [56]–[58] and dipeptidylpeptidase 4 (Dpp4) [59] function broadly in stem-cell pluripotency, homing, mobilization and immune responses and did not appear less abundant in our dataset. The lacking identification of transcripts but not the translated proteins may be caused by the unique membrane-protein recycling and trafficking processes via endosomes. [60]–[63] The fast dynamic membrane-protein cycling between the cell-surface membrane and endosomes is important in maintaining plasma membrane homeostasis [60], [61] and pattern formation (e.g. the polarization of endothelial cells) [64], which complements the relative slower responses at transcription and translation levels to variable extracellular micro-environments.

### N-glycosylation in E14.Tg2a Stem Cells

**Figure 5:**
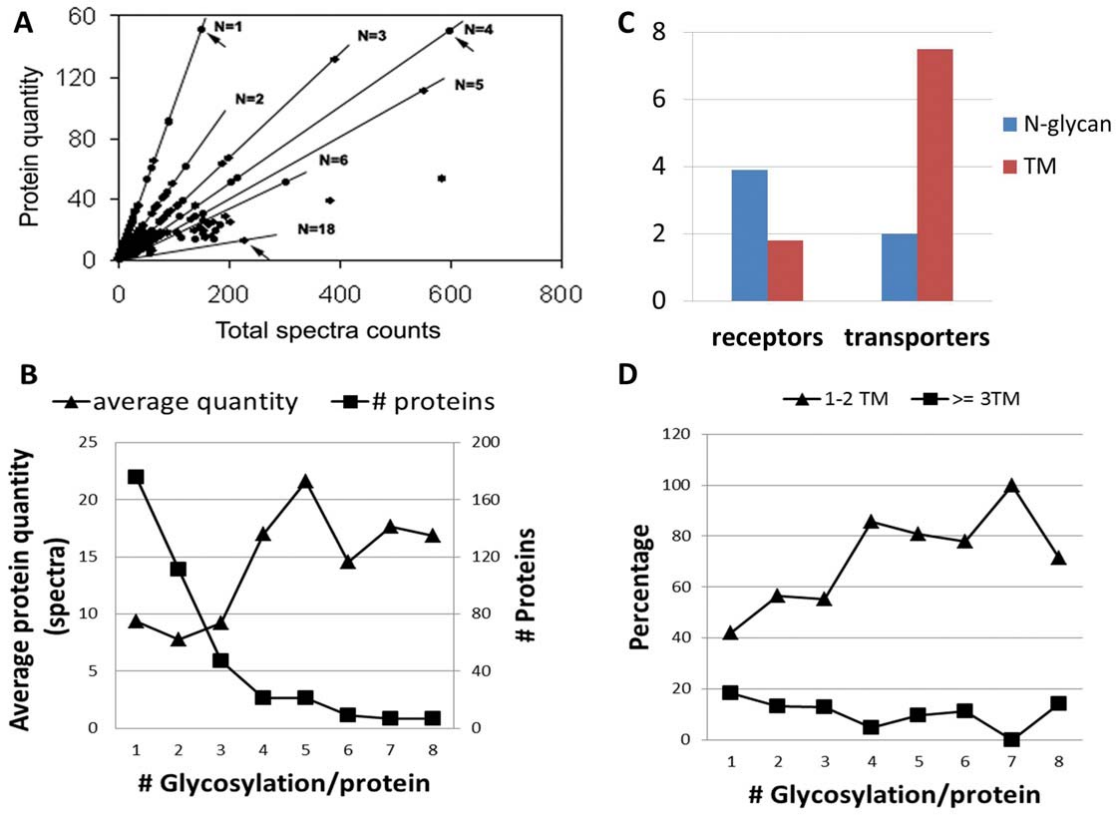
Quantitative analyses of detected N-glycoproteins and their N-glycan stoichiometries. A) A dotplot of protein spectra counts (X axis) over peptide spectra counts (Y axis) and the slope (N) is the number of N-glycosylation per protein (the N-glycan stoichiometry). Arrows point to Slc2a3 (N = 1), alkaline phosphatase (N = 4), and Lrp2 (N = 18). B) The average protein quantity and number of identified protein species as a function of protein glycosylation stoichiometry. C) Numbers of N-glycans and transmembrane (TM) domains in the identified receptors and transporters. D) The percentage of proteins with 1 and 2 TM vs. those with more than 3 TMs, as a function of protein glycosylation stoichiometry.

Using glycopeptide-capture approach, we characterized the N-glycoproteome of mES E14.Tg2a cells. A total of 1182 glycosites were identified from 3553 putative NXS/T sequons embedded in 468 glycoproteins (Table S1). The overall distribution of N-glycosylation stoichiometry as well as glycoprotein abundance is shown in Figure 5A. A few abundant proteins are highlighted in the figure by arrows. The average expression of the identified N-glycoproteins was about 10 spectra. Within these glycoproteins, the stoichiometry of N-glycosylation per protein ranges from 1 to 18, as indicated by the “N” value in Figure 5A with an average of 2.1 per protein (a ratio of the total number of identified N-glycosites over glycoproteins), and the average sequon occupancy was 44.4% (an average of individual protein sequon occupancy, which is a percentage of the detected glycosites over total number of sequons of each protein). The majority of the identified proteins (71%) exhibit a below-average expression; in fact, proteins with less than 4 protein spectra (low abundance by our definition) account for 44% of the total identification.

We grouped the glycoproteome based on the stoichiometry of protein glycosylation (shown in Table S5) and compared the average protein level and population size across groups with 1–8 glycans as shown in Figure 5B. In the figure, mono-glycosylated proteins constitutes close to half (43%) of the E14.Tg2a glycoproteome and the size of each group decreases exponentially with the increase of glycosylation stoichiometry. On the contrary, heavily glycosylated proteins (high stoichiometry) were expressed at higher level than less glycosylated proteins (low stoichiometry). From proteins with one glycan to those having eight glycans, the protein spectra count increases almost a fold from 9.3 spectra to 17 spectra; however, the protein population drops more than 20 fold from 174 to 7.

These results raise intriguing questions on what and why a small number of high-abundance and highly glycosylated membrane proteins exist. A functional search of the proteins with more than 8 glycans (Table S5) revealed that these glycoproteins carry important functions in stem cells. For instance, Lrp2 and Lrp1 (low-density lipoprotein receptor-related proteins) participate in receptor-mediated endocytosis and are critical to embryogenesis and nervous system development [65]–[67]. The interactome of Lrp2 has been recognized to be a variety of signaling molecules including sonic hedgehog, bone morphogenic protein 4, vitamin D-binding protein, retinol binding protein, and apo-lipoprotein E, among others that are important to stem cell biology [65]–[67]. Other high-abundance glycoproteins include Lama1 and Lama5, which are crucial for the attachment, migration, and organization of cells into tissues during embryonic development [68]. Along the same list is Tmem2, a functionally unknown protein, with a recent serendipitous discovery of its linkage to BMP4 signaling in cardiac development [69].

In search of why these functionally important proteins of stem cells are heavily glycosylated, we discovered to our surprise a complementarity between the glycosylation stoichiometry and the number of transmembrane (TM) domains. In Figure 5D, when we plot the percentage distribution of proteins with low TMs (≤2TM) versus high TM (≥3TM) of each glycosylation stoichiometry group, a decrease of high TM is observed with the increase of N-glycans on proteins. Such complementarity is more pronounced when we looked at the transporters and receptors based on the classification of Almen *et al.* (Figure 5C and Table S3). In detail, receptors carried on average 4 N-glycans/protein but less than 2 TMs, whereas transporters had one fold less N-glycans (2 per transporter on average) but two and half fold more TMs (7 per protein). More interestingly, sequons in these proteins differ also by one fold, i.e. 12 sequons per receptor and 6 sequons per transporter. Thus, both receptors and transporters in fact have similar glycosylation rate, i.e. 37.7% and 42.4% respectively, which is close to the average of the entire glycoproteome, i.e. 44.4%. Because both sequons and TMs can be predicted from protein sequences, we pondered whether we can observe similar compensation in other species from their protein sequences.

**Figure 6:**
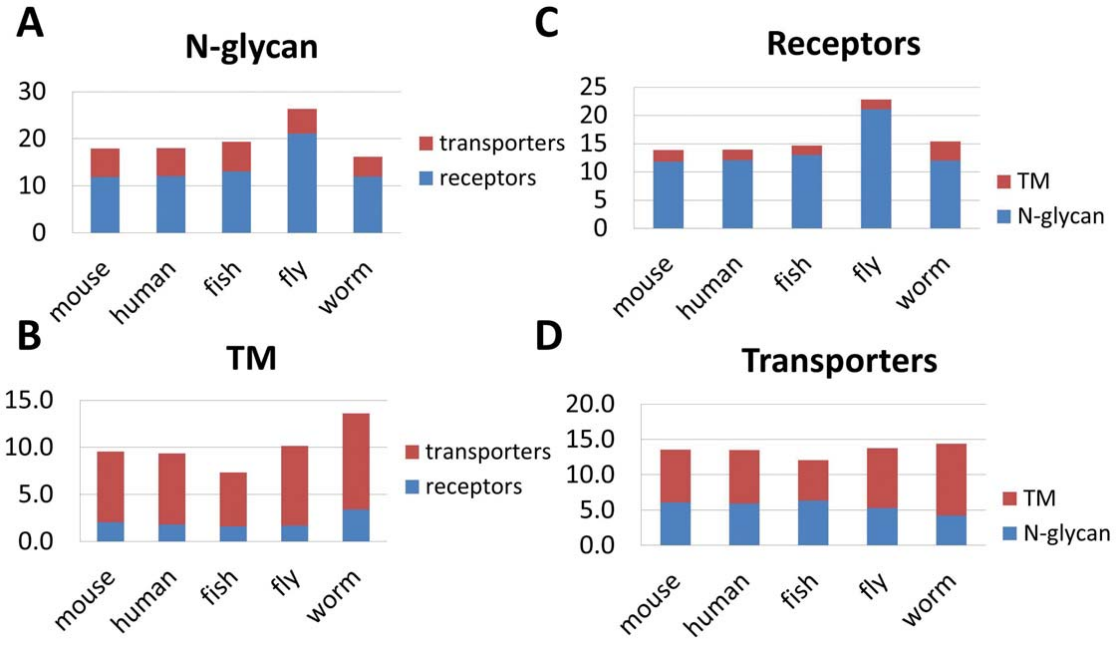
The number of predicted N-glycans and transmembrane (TM) domains based on protein sequences from listed species. Proteins are selected based on the sequence homology to the identified mouse proteins. Homology mapping is conducted NCBI HomoloGenes. Mouse: *Mus Musculus*; human: *Homo Sapiens*; fish: *Danio Rerio*; fly: *Drosophila Melanogaster*; worm: *Caenorhabditis Elegans*.

As a validation, we examined NXS/T sequons in orthogonal protein sequences derived from human (*Homo Sapiens*) as well as another three evolutionarily distinct species: fish (*Danio Rerio*), fly (*Drosophila Melanogaster*), and worm (*Caenorhabditis Elegans*) and the results are shown in Figure 6. The trend in the figure remains the same across all the species compared; thus, it appears that the complementarity we observed is determined by protein sequences and it is conversed cross different genera.

In a separate analysis, we looked a different class of glycoproteins, which function in glycobiogenesis pathways, i.e. glycosyl transferases and hydrolases. In our dataset, there are 9 glycosyl transferases and 14 glycosyl hydrolases (Table 2), and many of which were exclusively identified by our approach when comparing to global proteomic [52] or transcriptomic [51] analyses. More interestingly, this table reveals another occupancy pattern of sequons, i.e. all identified glycosyl transferases are monoglycosylated with a 28.1% glycosylation rate which is lower than the average (i.e. 44.4%); whereas most identified glycosyl hydrolases (10 of 14) contain more than two N-glycans with a more than one fold higher glycosylation rate of ∼58.7%. Nevertheless, the number of sequons buried in the sequences of these proteins are however very close, with glycosyl transferases and glycosyl hydrolases having (on average) 3.8 and 4.5 sequons respectively and the TM domains of all the listed proteins, if there is any, is 1 except Sttb3 that has 10 TMs (Table 2).

**Table 2:** Identified glycosyl transferases and hydrolases by the shotgun glycopeptide capture approach.

Gene ID	Gene symbol	TMs	Glycoprotein^c^	Detected glycosites	Putative glycosites
**Glycosyl transferases**					
**108105**	**B3gnt5**	1	2.0E+00	1	4
**54616**	**Extl3**	1	2.0E+00	1	4
**320011**	**Ugcgl1**	0	9.0E+00	1	3
**99151**	**Cercam**	0	3.0E+00	1	4
**68292**	**Stt3b**	10	5.0E+00	1	6
**223827**	**Glt8d3**	1	5.0E+00	1	3
**103963**	**Rpn1**	1	6.0E+00	1	2
**234407**	**Glt25d1**	0	2.4E+01	1	3
**56386**	**B4galt6**	1	4.0E+00	1	9
**Glycosyl hydrolases**					
**12182**	**Bst1**	1	3.0E+00	1	4
**14387**	**Gaa**	1	7.5E+00	4	7
**14667**	**Gm2a**	0	2.0E+00	1	1
**110006**	**Gusb**	1	1.5E+00	2	4
**17159**	**Man2b1**	0	2.0E+00	3	11
**14466**	**Gba**	0	3.1E+01	2	5
**17939**	**Naga**	0	2.5E+00	2	3
**100340**	**Smpdl3b**	0	5.5E+00	2	5
**12494**	**Cd38**	1	2.1E+01	4	4
**11605**	**Gla**	1	7.0E+00	1	3
**17156**	**Man1a2**	1	3.0E+00	1	1
**17158**	**Man2a1**	1	2.0E+00	3	3
**66967**	**Edem3**	1	1.5E+01	1	7
**15587**	**Hyal2**	0	1.8E+01	3	5

### Power to Identify Stem Cell Surface Markers

**Figure 7:**
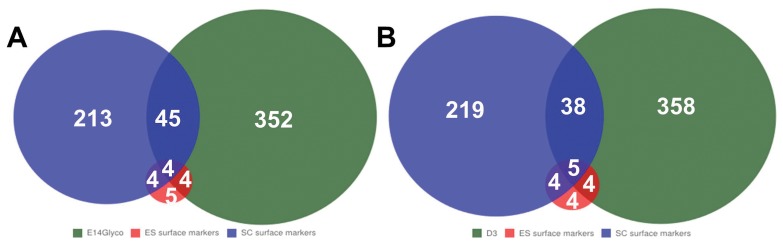
Comparison of N-glycoproteome and cell-surface membrane proteome in the identification of ES-cell surface markers. A) Comparison between our N-glycoproteome (E14Glyco), the curated known ES and the somatic stem-cell markers. B) Comparison between cell-surface membrane proteome (D3) [22] and the same stem-cell marker sets.

Because of the unique localizations of N-glycoproteins, we were able to identify cell surface proteins effectively. For example, we detected a large number of CD proteins (Table 1), and known embryonic and somatic stem-cell markers (Figure 7). For the number of markers identified, our method performed similarly as surface proteomics. The inefficient disclosure of known stem-cell markers is alike the incomplete identification of proteins validated by immunoblotting assays in Figure 3C & 3D, indicating that the analytical sensitivity of both methods needs to be improved.

## Discussion

### Importance and Challenges of Studying ES Membrane Proteins

Stem cells possess unique properties and broad medical applications. Understanding and harnessing these almighty cells have a profound impact in developmental biology, regenerative medicine and personalized therapies [1], [2]. Membrane proteins help mediate the communication between stem cells and their immediate environments (including neighboring cells), thereby playing pivotal roles in stem-cell biology. The characterization of membrane proteins allows functional understanding and a potential control of these molecules. In addition, a proteomic-scale analysis enables holistic views of the cell surface, in which different yet simultaneously happening processes and functions can be examined together and novel synergies can be discovered.

Even though large-scale genomic analyses have greatly advanced our understanding of stem cells [70]–[72], direct protein analyses are necessary and important for numerous reasons. One of the important reasons is that many protein functions are regulated by post-translational processes, which are beyond the scope of genomics. We therefore used proteomic approach and characterized in the current study proteins with N-glycosylation, one of the most structurally complex co- and post-translational modifications in biological systems. The unique cellular localization of N-glycoproteins allowed us to effectively identify cell surface proteins when the results were compared with other membrane proteomes (Figures 3 & 4). In addition, our results (Tables S2 and S5) described the sites and stoichiometry of protein N-glycosylation at proteomic scale, enabling the building of novel links between protein structure and function that genomic studies cannot provide.

Membrane proteomics have been challenged by high hydrophobicity, heavy modification, and low expression of membrane proteins [15], [16]. We showed here that membrane-targeted proteomics including our N-glycoproteomics is capable of improving the characterization of stem-cell surface proteins compared to transcriptomics and whole cell proteomics (Figure 3 & 4). The difference between our strategy and other membrane proteomics is the targets of enrichment. Instead of isolating cell plasma membrane or cell outer-surface proteins, we have chosen to enrich N-glycoproteins.

The unique localizations of N-glycans, i.e. cell external surface and extracellular space as well as the secretary pathway, define our N-glycoproteome as a combination of ectoproteome and endosecretome (i.e. proteins of the endomembrane system). Because of the dynamic relationship built between cell surface and endomembrane system through endocytosis and exocytosis processes [62], [63], we believe that the endosecretome can be viewed as an extended ectoproteome. From the experimental results in Figure 3, regardless the designated targets proteins both ecto- and endo-membranes are enriched in all surface-membrane enrichment strategies, and there is no significant difference between our N-glycoproteome and other membrane proteomes as regards the membrane protein identification. Therefore, we argue that it is molecularly and biologically reasonable to expand the physiological plasma membrane to the endo-membrane systems downstream until the ER.

What mostly discerns our results from the rest however is not the inclusion of endomembrane system, but the exclusion of the abundant cytosolic proteins, such as ribosomal proteins. The unexpected appearance of cytosolic proteins in several surface proteomic results may be caused by plasma-membrane recycling processes such as pintocytosis that can internalize labeling reagents and potentially lead to the labeling and subsequent identification of cytosolic proteins. Under perturbations, such as xenobiotics, cellular endocytosis processes can be enhanced [62], [63], and labeling reagents are xenobiotics to living cells. Targeting to N-glycosylation allows us to avoid the labeling of live cells, and the hydrazide chemistry we employed forms chemical bonds between glycoproteins and the capture resin, such that cytosolic proteins can be easily removed by stringent washes. Thereby, our result appears to have the least cytosolic protein contamination.

### The Landscape of Membrane Proteins of mES E14.Tg2a

Using low-end instruments but a sensitive glyco-peptide capture technique, we were able to profile for the first time the N-glycoproteome of E14.Tg2a mouse embryonic stem (mES) cells (Tables S1 & S2). The molecular information presented here materializes our conceptual understanding of membrane proteins regarding to the low quantity yet high diversity. Among all the identified membrane proteins, receptors are closely resembled the property of low quantity yet high diversity of membrane proteins (Figure 2 and Table S3), which can be tied with their highly regulated sensing and signaling functions. On the contrary, transporters are relatively abundant which may be a requirement or consequence of the fast growth of mES cells *in vitro*. From a cross-validation of our findings with other previously published cell surface proteomes in Figures S2A & S2B in File S1, we observed similar trend except in red blood cells (RBCs). For RBCs, we observed an opposite trend with low number of receptors but high number of transporters. Considering RBCs function heavily in oxygen exchange, the identification of more transporters than receptors in RBCs is reasonable. It will be interesting to look at these findings from a much broader view when more and more surface proteome datasets becomes available. For E14.Tg2a cells, the defect of HPRT is known to interfere the purinergic receptor expression on the cell surface [73], and purinergic receptors are ATP gated ion channels that function in stem cell proliferation and differentiation [57], [58]. Our method successfully identified this receptor family, therefore, can be a potential tool to study the pathophysiology of the Lesch-Nyhan syndrome.

A specific question raised from our result is whether the observed diversity of membrane proteins is specific to E14.Tg2a mES or generic to all cells. Several findings support that the stem-cell membrane proteome may be more diversified than those of specialized cells. For example, the most abundant protein in our data is a glucose transporter, Slc2a3 with an estimated quantity of 10,000 copies per cell, which is one hundred times lower than the copy number of the most abundant membrane protein – anion exchanger 1 (1 million copies/cell) [44], [74] in functionally specialized red blood cells. Even though our value is estimated from a single reference, Lifr, the large discrepancy with potential variations can still provide us some insights of the protein dynamic range and complexity at these cells’ surfaces. Another reason for us to believe the detected diversity at least partially specific to stem cells is the population heterogeneity inherent to stem cells [75], which increases the likelihood to observe different membrane proteins. For example, the examination of the proteomes of 7 mES cell studies, each with >1000 protein identifications, yielded only a small overlap of 27% among themselves; and about 61% of the proteins were identified only in one or two studies [75].

### Classification of Cell Types

One major advantage of molecular profiling of biological systems is to classify these systems for similarities and differences at molecular level for functional implications. Similar to phylogenetic tree built upon genome sequencing from which we can evaluate the evolutionary distance of species, a dendrogram built upon surface proteins can allow us to distinguish hidden relationships among different cell types, developmental stages, and cultured environments. Using commonly identified proteins in 48 human tissues and 45 human cell lines, Uhlen’s group was able to classify cell, tissue and organ types [76]. We expect the classification based on surface proteins will be more sensitive than those based on total proteins, because whole-cell proteomes will more likely be overwhelmed by abundant housekeeping proteins than surface proteomes, and abundant proteins contribute less to the classification than cell specific proteins.

From our data, we noticed that a classification using GO differential enrichment (Figures 3 and 4) can offer insights not only to the cell types, such as mES cells vs. RBCs, but also to the different techniques used to derive these surface proteins. If applying different techniques to the same cells, we anticipate a more detailed characterization of closely related techniques.

### N-Glycosylation in E14.Tg2a mES Cells

In addition to the identity of glycoproteins, our method also provides experimental evidence to proteins’ sites of N-glycosylation. Denoting the site of N-glycosylation is important to study protein structure and function, because N-glycosylation play key roles in assisting protein folding and trafficking, protecting proteins from enzymatic degradation, and participating frequently in binding events such as those in signaling and immune responses [11], [77]. The prevailing N-glycosylation had only been studied on individual proteins before high-throughput molecular characterization became available; therefore the knowledge of N-glycosylation is fragmentary. Several databases curate known protein N-glycosylation; however, much effort is still needed to comprehend these collections. For example, in Swiss-Prot database about two-thirds of entries containing NXS/T sequons, but only 16% of these entries are filed as glycoproteins and only 1% have been characterized with respect to the site of attachment and the nature of the carbohydrate unit [78]. In our study, we mapped 400 out of 405 identified genes to 652 Swiss-Prot proteins (2012_08 release), and only 152 of the latter are annotated as glycoproteins in the database. Our method, therefore, is efficient in providing experimental supports to glycoproteins and their sites of glycosylation.

When we deliberately checked a few previously characterized glycoproteins, a good match was observed. For example, Rpn1 (ribophorin 1), which is part of the oligosaccharyltransferase complex, has four putative glycosylation sites, but only the NVS motif at Asn276 was shown to be glycosylated in previous biochemical studies [79]. Bst1 (bone marrow stromal cell antigen 1) also has four putative N-glycomotifs; site-directed mutagenesis suggested that the fourth motif at Asn192 is N-glycosylated and is important for maintaining cyclic ADP ribose hydrolase activity. [80] Our results support both of the findings. In another case, for purinergic receptor ATP-gated ion channel, the N-glycosylation has been demonstrated to control ATP binding [81]. Yet for subtypes of purinergic receptors, multiple glycosylation sites exist but no experimental evidence has been provided on their exact glycosylation patterns. Our results offer the occupancy to all the sequons, leading to a confirmation of their membrane orientation in mES cells, as N-glycans selectively localized to the external site of a cell.

A quantitative examination of N-glycosylation and transmembrane (TM) domains in the N-glycoproteome allowed us to discover an interesting complementary relationship between the numbers of N-glycans and TMs on receptors and transporters defined by Almen *et al.* [40] as shown in Figure 5C. Two factors determined N-glycan stoichiometry, one is the number of sequons in protein sequences, and the other is the rate of N-glycosylation which is largely regulated by the N-glycogenesis pathway in the ER and Golgi. As the examined receptors and transporters shared the similar glycosylation rate, we hypothesized that the observed glycopattern is determined by the number of sequons in protein sequences. As the TM domain is also determined by protein sequences, it is therefore possible to verify the observed offset between the numbers of TM and N-glycans by protein sequences. A further sequence analysis (Figure 6) in four evolutionarily distinct species, i.e. human (*Homo Sapiens*), fish (*Danio Rerio*), fly (*Drosophila Melanogaster*), and worm (*Caenorhabditis Elegans*), besides mouse (*Mus Musculus*) confirmed our hypothesis.

If the complementarity we observed is evolutionarily conservative, it may be critical to protein stability and function. Obviously, TMs help stich proteins into the cell membrane, and it is also known that cell surface glycosylation helps shield proteins from attacks of degradation enzymes. The adoption of some protections by the form of TMs and N-glycans in membrane proteins seems natural. It is also reasonable for us to hypothesize that the increased protection of one form can decrease the need of the other, then how do proteins make a choice between the two: TMs versus N-glycans, since apparently for receptors and transporters, there is a preference in such decision? We sought answers from the glycosylation process. N-glycosylation is known as a co-translational modification occurring *en bloc* to the newly synthesized polypeptides on the inner surface of ER lumen through membrane-bound oligosaccharide transferases [82]. The accessibility of sequons to the transferase will affect the success of glycosylation [83]. Early work of von Heijne’s and Reithmeier’s groups [83], [84] has discovered that a space with minimum distance of 12–14 amino acids is required for N-glycosylation to happen in the ER lumen. Thus, we believe N-glycosylation is physically limited from proteins with high TM domains.

If so, we are puzzled by the factors that select one protection over the other and form a pattern appeared on receptors and transporters as shown in Figures 5C & 6. Even though both TM and glycosylation can protect membrane protein integrity; being different from TMs, N-glycosylation is an additional modification to the poly peptide chain, and N-glycans are typically bulky in size which are expensive to synthesize in terms of energy, however the synthesized N-glycans can carry multiple functions. The chunky N-glycans not only are the major constituents of cell walls and membrane glycocalyx for protection mechanisms, they also frequently participate in binding events to facilitate cell-cell and cell-matrix communication [85], immune responses, and signaling events. Both receptors and transporters need to interact with other molecules; however, the molecular interactions of receptors need to be specific and tightly regulated, whereas those of transporters require moving the cargos in and out swiftly. Given the fact that receptors tend to be low in abundance, the usage of fewer TMs but more glycans for receptors to protect themselves may functionally favor in their need of modulated binding specificity and efficiency to their targets. Even though glycan binding is weaker than antibody antigen binding, the branched glycans can provide multiple binding sites thus increasing the overall avidity [86]. In retrospect, the preference of TMs over N-glycans in reporters may be a result of avoiding the blockage of cargo path by the chunky, extruded and rigid glycans. Therefore, the observed selection between TMs and N-glycosylation for protection of membrane proteins is likely determined by proteins’ functions. The built in glycosylation correlation in protein sequences makes it robust to environmental perturbations.

Yet, not all protein glycosylations are such a case. Another interesting glycol-pattern we discovered is from identified enzymes catalyzing glycobiogenesis, including glycosyl transferases and hydrolases. Different from transporters and receptors having similar glycosylation rates, transferases have much lower glycosylation rate (27%) as that of hydrolases (50%). A check of the cellular localization of these proteins indicates transporters and receptors that are mostly localized in similar microenvironments; whereas glycoenzymes reside disparately in cellular organelles, with glycotransferases preferentially mature in the secretory pathway, represented by the ER and Golgi, while hydrolases mature in the endocytic pathway, represented by lysosomes and endosomes. The microenvironments regarding the number of proteolytic enzymes are distinct in these two pathways; therefore the need of protection is stronger for glycohydrolases than glycotransferases. We hypothesize that the observed glycosylation discrepancy is likely influenced by the micro-environment for protection needs.

Experimentally, imaging analyses have discovered that lysosomal inner membranes are covered by glycocalyx [87], a structure that has been observed at mammalian cell surface to protect cell integrity. It is not surprising to see lysosomes adapt the similar means to protect their membrane proteins as the lysosome lumen has harsher condition than extracellular space regarding the variety of hydrolases. A removal of the N-glycans from abundant lysosomal proteins, Lamp 1 & 2, has decreased the stability of these proteins [88], [89]. To further validate our hypothesis, we examined the glycosylation rate and localization of all the identified proteins. Besides the glycosyl hydrolases, we also discovered extracellular matrix proteins that are heavily glycosylated with few TMs. Taken together with the complementarity built in transporters and receptors, we hypothesize from our observation an evolutionarily conserved and environmentally influenced fitness or adaptation mechanism represented by N-glycosylation for membrane proteins to achieve appropriate functions. It will be interesting to test our narrative in the broader contexts for the prevalence and specificity of such evolutionary and environmental causality on proteins’ structure and function.

### Sensitivity of the Glycopeptide-capture Strategy

Because we used ordinary instrument, the apparent sensitivity of our analysis can be readily improved. Several analysis results such as those shown in Figures 3C, 3D, and 7 have indicated such necessity. For curiosity, we estimated our detection sensitivity by correlating the known quantity of Lifr on mES cells (∼100 copies/cell) to our spectra counting results ∼ 4 spectra. In such case our single-spectra identification correlated to glycoproteins with about 20–30 copies per cell. As we used 3×10^8^ cells, considering sample loss and multiple instrumental replicates during sample processing and detection, the sensitivity of detection is roughly about femtomole. This result agrees the generally accepted instrumental sensitivity. Thus, we estimate our detection sensitivity to be below 100 protein copies. If more absolute protein quantity information available, our estimation should be more accurate. Current high-end MS instrumentation can easily achieve sub-femtomole to tens of attomole detection sensitivity. Coupling our enrichment method with these fast and accurate MS with nano-LC with more exquisite flow control and high pressure range, we are expecting a substantial improvement in the results.

### Conclusions

We characterized the N-glycoproteome of E14.Tg2a including a list of functionally important receptors, transporters, enzymes, CD molecules and stem cell markers. Our method demonstrates the same, if not better, coverage of cell surface proteins as those of other membrane proteomics. The obtained N-glycoproteome also includes proteins residing in endomembrane compartments, which we consider to be the extended surface membranes. Our results and analyses provide, for the first time, a global yet quantitative topology of E14.Tg2a membrane proteins: a 1∶2 protein species ratio and a 2∶1 quantitative ratio of transporters to receptors; an average membrane-protein quantity of ∼ 10 spectra (equivalent to ∼ 2×10^2^ copies/cell). Majority of identified proteins (>70%) have below average quantity; and the most abundant N-glycoprotein in the E14.Tg2a is the solute carrier protein Slc2a3, and the most N-glycosylated protein is a low-density lipoprotein receptor like protein, Lrp2. For N-glycosylation, we noticed an average glyco-occupancy of 33% and an average stoichiometry of 2.1 N-glycans/protein.

Besides such global statistic numbers, the most intriguing discovery we made is an evolutionary selection of N-glycosylation over the transmembrane unit built into protein sequences to stabilize membrane proteins and to facilitate their functions, which can be robust to environmental perturbations as in the cases of transporters and receptors, or be flexible to the immediate microenvironments such as the cases of glycoenzymes and extracellular proteins.

Using ordinary LC-MS, we demonstrated here the power of a high-throughput proteomics in studies of post-translational modification from a systematic viewpoint, and we hope that the obtained N-glycoproteome of E14.Tg2a will help a better understanding of the molecular background of this important cell line.

## Supporting Information

Table S1 **The total identified peptides, proteins and glycoproteins.**
(XLS)

Table S2 **The identified glycoproteome and their expression quantity characterized by spectra counting.**
(XLSX)

Table S3 **Classes of identified glycoproteins based on the classification published by Almen** ***et al.***
(XLSX)

Table S4 **Gominer annotation of glycoproteins uniquely identified in our results than those obtained from the global characterization of the transcriptome and proteome.**
(XLSX)

Table S5 **List of identified N-glycoproteins based on their number of N-glycosylation.**
(XLSX)

File S1 **Supplementary material including supporting text, figures and references.**
(DOC)
