# Supplementary material for: N-Glycoproteome of E14.Tg2a Mouse Embryonic Stem Cells
Source: PLoS One. 2013 Feb 6;8(2):e55722. doi: 10.1371/journal.pone.0055722 (PMC3565968; doi:10.1371/journal.pone.0055722)
Supplement: File S1 — Supplementary material including supporting text, figures and references. (DOC) [file pone.0055722.s006.doc]

**Supplementary Materials**

**Mass spectrometry analysis of glycopeptides**

For LTQ mass spectrometry analysis, an in-house fabricated nanoelectrospray source and an HP1100 solvent delivery system (Agilent, Palo Alto, CA) with split flow were coupled to LTQ. Samples were automatically delivered by a FAMOS autosampler (LC Packings, San Francisco, CA) to a 100 μm internal diameter fused silica capillary pre-column packed with 2 cm of 200 Å pore-size Magic C18AQ™ material (Michrom Bioresources, Auburn, CA) as described elsewhere .

The samples were washed with solvent A (5 % acetonitrile in 0.1 % formic acid) on the pre-column and then eluted with a gradient of 10-35% solvent B (100% acetonitrile) over 30 minutes to a 75 μm x 10 cm fused silica capillary column packed with 100 Å pore-size Magic C18AQ™ material (Michrom) and then injected into the mass-spectrometer at a constant column-tip flow rate of ~300 nL/min. Eluting peptides were analyzed by nanoLC-MS and data-dependent nanoLC-MS/MS acquisition, selecting the 3 most abundant precursor ions for MS/MS with a dynamic exclusion setting of 1 repeat counts, 30 seconds repeat duration, and 180 seconds exclusion duration with an exclusion list size of 50 .

**Evaluation of the proteomics analysis**

The glycopeptide-capture was applied three times to different mouse embryonic stem (mES) cell samples (biological replicates), and each captured peptide mixture was analyzed in triplicates by the LC‑MS/MS (technical replicates). Two biological replicates have further separated by offline strong-cation-exchange (SCX) chromatography prior to reverse-phase nano-LC-MS/MS analysis.

We evaluated the sensitivity gained by the additional SCX separation as shown in Figure S1, in which a dot plot records the quantitative comparison of protein spectra counts between two separation schemes, i.e. with and without off-line SCX. The uniquely identified proteins by one approach are on the corresponding axes, and the commonly identified proteins are in the quadrant of the plot. A linear correlation is resolved with R^2^ ≈ 0.8, indicating a good consistency between the two types of LC-MS analyses. The correlation coefficient is 0.34 suggesting a 3-fold improvement of detection sensitivity with SCX than those without.

**Evaluation of receptors and transporters relationship using other publically available datasets**

In order to evaluate the observed about one-fold difference between the total number of transporters and receptors, and another one-fold difference between the average spectra counts of these two classes of proteins, we further analyzed a few additional datasets, including Wollscheid’s and Nunomura’s mES surface proteomes as well as the red blood cell (RBC) surface proteome. The results are shown in Figure S2A and S2B. The qualitative relationship (Figure S2A) has preserved across all the compared datasets, including three mES cell datasets, one dataset from mES derived neuron precursor (NP) cells, one dataset from mES derived embryoid body (EB) cells, except the one dataset derived from RBCs (arrow pointed). For RBC, we observed an opposite trend with low number of receptors but high number of transporters. Considering RBCs function heavily in oxygen exchange, the identification of more transporters than receptors in RBCs is reasonable.

For the quantitative relationship, only our N-glycoproteome and the surface N-glycoproteomes are used for comparison because other datasets do not have quantitative information available. All the compared proteomes follow into a similar trend as shown in Figure S2B below, in which the average expression of receptors is lower than that of transporters. These agreements validate our results and also indicate that the trend we observed is presented by many different cell types, but differences do exist as shown by RBC cells.

**Quantitative comparison of commonly identified proteins between our N-glycoproteome and cell surface N-glycoproteome.**

Because both of these two datasets include quantitative information, it is possible for us to perform a quantitative comparison at the individual protein level between these two datasets (Figure S3). In the figure, except for the two abundant proteins (arrow pointed) seem quite off, the rest proteins fall in a pretty good agreement with our dataset, and our results provides a greater abundance range as most data points leaning towards x axis with larger values, thereby, our results can better distinguish the quantitative difference among N-glycoproteins. The inclusion of more low-abundance proteins into comparison renders the total trend less viewable, so we plotted them separately in Figure S3.


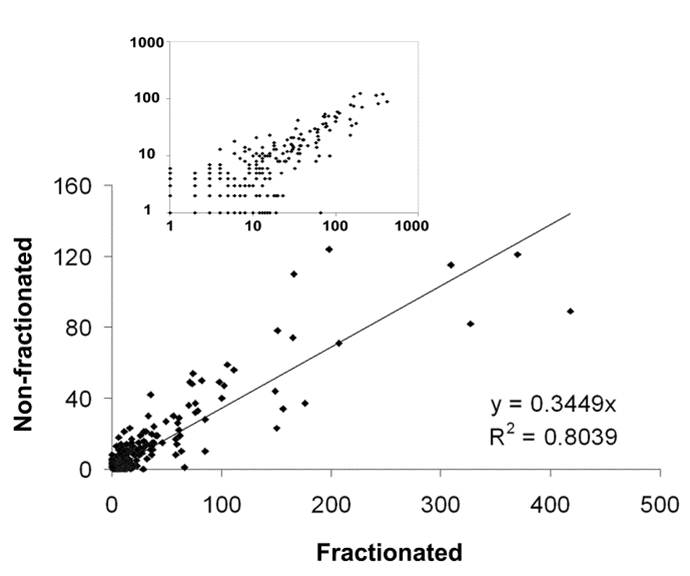
**Figure S1. The quantitative correlation of identified proteins between SCX fractionated and non-fractioned samples.** The quantity of the fractionated samples is the total peptide spectra counts of 2 biological replicates (each has 6 SCX fractions and 3 technical replicates); and the quantity of non-fractionated samples is the total peptide spectra counts of three biological replicates and each with 3 technical replicates. The probability cutoff of identified proteins is 0.9. A linear correlation exists with R^2^ of 0.80 and a coefficient of 0.34. The insert is a log plot for better visualization in the low spectral counts region.


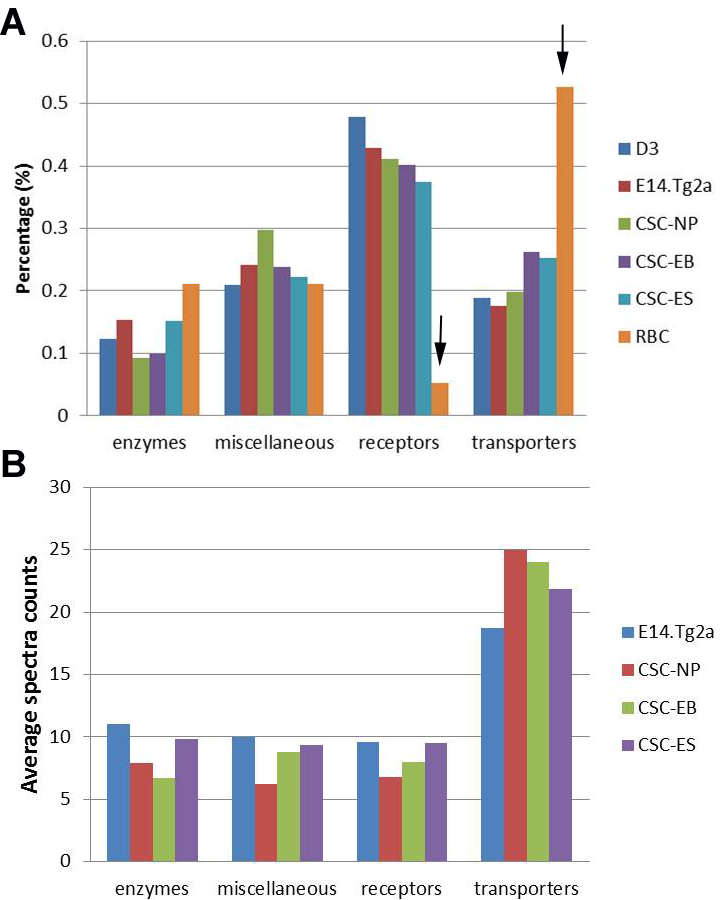
**Figure S2**. **Comparisons of specified protein classes based on Almen *et al.*’s cataloging among different surface protein datasets.** A, a comparison of the percentage of each protein class among different datasets; B, a comparison of the average spectra counts of each protein class in different datasets. D3 is a mES cell surface proteome, and E14.Tg2a is our mES cell N-glycoproteome. CSC represents the surface N-glycoproteomics method (Wollscheid *et al.*), and the results obtained by applying this method is separately represented by the analyzed cell types. NP and EB stand for the neuronal precursor cells and the embryoid body, respectively; ES refers to the mES cells used to derive both of the above mentioned NP and EB cells. RBC stands for a surface proteome obtained from red blood cells.


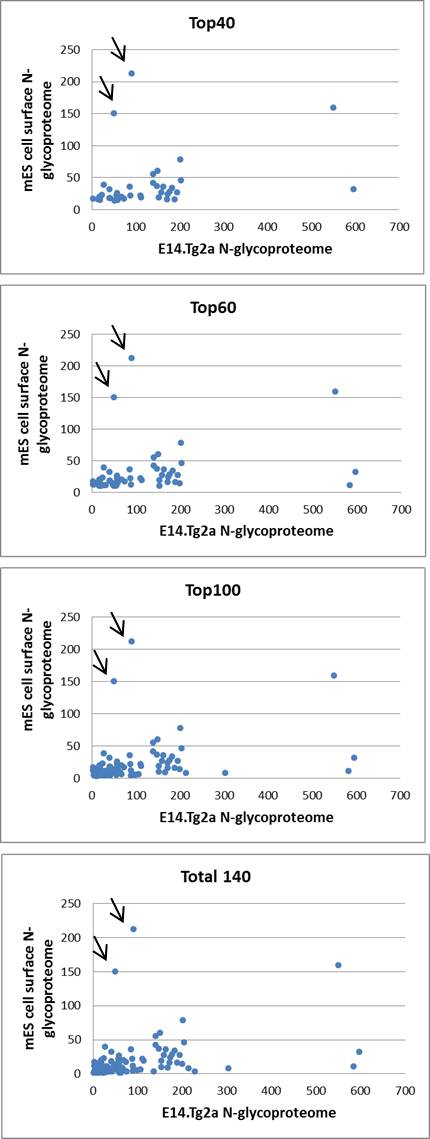
**Figure S3**. **The quantitative comparison of the abundance of commonly identified glycoproteins between the mES surface N-glycoproteome and E14.Tg2a mES N-glycoproteome.** The ranking from top 40 till top 140 is based on protein abundance presented in the surface N-glycoproteome. Arrows point to outliers.

**References:**

1. Yi EC, Lee H, Aebersold R, Goodlett DR (2003) A microcapillary trap cartridge-microcapillary high-performance liquid chromatography electrospray ionization emitter device capable of peptide tandem mass spectrometry at the attomole level on an ion trap mass spectrometer with automated routine operation. Rapid Commun Mass Spectrom 17: 2093-2098.

2. Gygi SP, Rochon Y, Franza BR, Aebersold R (1999) Correlation between protein and mRNA abundance in yeast. Mol Cell Biol 19: 1720-1730.

3. Cox B, Kislinger T, Emili A (2005) Integrating gene and protein expression data: pattern analysis and profile mining. Methods 35: 303-314.

4. Gao J, Friedrichs MS, Dongre AR, Opiteck GJ (2005) Guidelines for the routine application of the peptide hits technique. J Am Soc Mass Spectrom 16: 1231-1238.

5. Ishihama Y, Oda Y, Tabata T, Sato T, Nagasu T, et al. (2005) Exponentially modified protein abundance index (emPAI) for estimation of absolute protein amount in proteomics by the number of sequenced peptides per protein. Mol Cell Proteomics 4: 1265-1272.

6. Lu P, Vogel C, Wang R, Yao X, Marcotte EM (2007) Absolute protein expression profiling estimates the relative contributions of transcriptional and translational regulation. Nat Biotechnol 25: 117-124.

7. Rappsilber J, Ryder U, Lamond AI, Mann M (2002) Large-scale proteomic analysis of the human spliceosome. Genome Res 12: 1231-1245.

8. Wollscheid B, Bausch-Fluck D, Henderson C, O'Brien R, Bibel M, et al. (2009) Mass-spectrometric identification and relative quantification of N-linked cell surface glycoproteins. Nat Biotechnol 27: 378-386.

9. Nunomura K, Nagano K, Itagaki C, Taoka M, Okamura N, et al. (2005) Cell surface labeling and mass spectrometry reveal diversity of cell surface markers and signaling molecules expressed in undifferentiated mouse embryonic stem cells. Mol Cell Proteomics 4: 1968-1976.

10. Pasini EM, Kirkegaard M, Mortensen P, Lutz HU, Thomas AW, et al. (2006) In-depth analysis of the membrane and cytosolic proteome of red blood cells. Blood 108: 791-801.
